# Neuronal Prosurvival Role of Ceramide Synthase 2 by Olidogendrocyte-to-Neuron Extracellular Vesicle Transfer

**DOI:** 10.3390/ijms24065986

**Published:** 2023-03-22

**Authors:** Álvaro Casadomé-Perales, Sara Naya, Elisa Fernández-Martínez, Bea G. Mille, Marta Guerrero-Valero, Héctor Peinado, Francesc X. Guix, Carlos G. Dotti, Ernest Palomer

**Affiliations:** 1 Molecular Neuropathology Unit, Physiological and Pathological Processes Program, Centro de Biología Molecular Severo Ochoa, CSIC/UAM, 28049 Madrid, Spain; 2 Microenvironment and Metastasis Group, Molecular Oncology Program, Spanish National Cancer Research Centre (CNIO), 28029 Madrid, Spain; 3 Department of Bioengineering, Institut Químic de Sarrià (IQS), Universitat Ramón Llull (URL), 08017 Barcelona, Spain

**Keywords:** brain ageing, EVs, exosomes, CerS2, intercellular communication, oligodendrocyte-to-neuron

## Abstract

Ageing is associated with notorious alterations in neurons, i.e., in gene expression, mitochondrial function, membrane degradation or intercellular communication. However, neurons live for the entire lifespan of the individual. One of the reasons why neurons remain functional in elderly people is survival mechanisms prevail over death mechanisms. While many signals are either pro-survival or pro-death, others can play both roles. Extracellular vesicles (EVs) can signal both pro-toxicity and survival. We used young and old animals, primary neuronal and oligodendrocyte cultures and neuroblastoma and oligodendrocytic lines. We analysed our samples using a combination of proteomics and artificial neural networks, biochemistry and immunofluorescence approaches. We found an age-dependent increase in ceramide synthase 2 (CerS2) in cortical EVs, expressed by oligodendrocytes. In addition, we show that CerS2 is present in neurons via the uptake of oligodendrocyte-derived EVs. Finally, we show that age-associated inflammation and metabolic stress favour CerS2 expression and that oligodendrocyte-derived EVs loaded with CerS2 lead to the expression of the antiapoptotic factor Bcl2 in inflammatory conditions. Our study shows that intercellular communication is altered in the ageing brain, which favours neuronal survival through the transfer of oligodendrocyte-derived EVs containing CerS2.

## 1. Introduction

Age-associated cognitive deficits are caused mainly by the reduced ability to encode new episodic and factual memories, the use of information to execute tasks (working memory) and the processing speed of information. At the anatomic/structural level, these age-related changes are associated with a decline in both grey and white matter volume [1,2,3,4,5,6,7]. Volume changes seem to arise from reduced dendritic arborisation, dendritic spines and synaptic contacts rather than cell death [8,9,10,11]. This fact can be observed as a sacrifice of neuronal function in favour of neuronal survival.

Excluding very advanced age, most cognitive decline in ageing occurs gradually and does not significantly impact basic brain functions due to the central nervous system’s robust capacity for molecular and cellular adaptation throughout life. In physiological ageing, these adaptative capacities preserve the neural circuitry required for sensing, storing and processing information from seconds to years. Although a large body of evidence focuses on neuronal plasticity in ageing (changes in neuronal structure and synaptic strength), a number of studies suggest that myelinating activity is also highly plastic during ageing. This plasticity is evidenced in the generation of new myelinating cells, in myelin sheath length, thickness and even in the distribution along axons. Electron microscopy studies demonstrated redundant myelin formation in ageing, evidenced by a double set of sheaths where one set is surrounded by a second set of compact lamellae [12]. In line with myelin plasticity with age, studies in primates revealed an increase in oligodendrocyte and microglia numbers with age [13,14]. A similar conclusion was drawn about the neocortex of the ageing rat [15]. An increase in gliogenesis and myelinogenesis was also demonstrated as a function of age in the spinal cord [16]. Altogether, these results strongly support the notion that part of the nervous system’s plastic capacity to adapt to age-associated adverse effects relies on the activation of myelination mechanisms.

In recent years, intercellular communication mechanisms via extracellular vesicles (EVs) have been extensively associated as a mediator of myelogenesis. The term EV includes two types of lipid bilayer vesicles released into the extracellular space: exosomes (40–160 nm size and formed by invagination of different biomolecules in compartments called multivesicular bodies) and microvesicles (100–1000 nm size and formed by direct fusion of the content with the plasma membrane) [17]. Numerous studies have shown that neurons communicate with neurons [18], astrocytes [19], microglia [20] and oligodendrocytes [21] through the secretion of EVs.

Ageing alters many cellular physiological mechanisms, including EV-mediated communication mechanisms. A clear decrease in the number of EVs released into the cerebrospinal fluid has been observed in healthy human old subjects [22], and in Alzheimer’s disease (AD) murine models [23]. In addition, a growing body of evidence points to a change in the content of EVs in ageing, as observed for proteins such as Tau p181 or amyloid-β (Aβ)42 (related to AD), cathepsin D (lysosomes) or neurogranin (prion disease) [24,25]. Additionally, changes in EVs’ content of micro-RNAs such as miR-23a-5p or miR-137, which can promote senescence [26,27] were observed.

Due to all of the above, we investigated the implication of EVs in neuronal death-survival balance during ageing. First, we examined the content of EVs isolated from young and old brains by proteomics. This study revealed the existence of changes in numerous metabolic pathways, the most notable being in the vascular system, autophagy, mitochondrial function, calcium homeostasis, epigenetics and oligodendrocyte function. In this work, we focus on the analysis of an oligodendrocyte protein that increases significantly in cortical EVs with age: ceramide synthase 2 (CerS2).

## 2. Results

### 2.1. Age Leads to a Reduced Number of Cortical Extracellular Vesicles, Which Are Enriched in the Oligodendrocyte Enzyme CerS2

**Figure 1:**
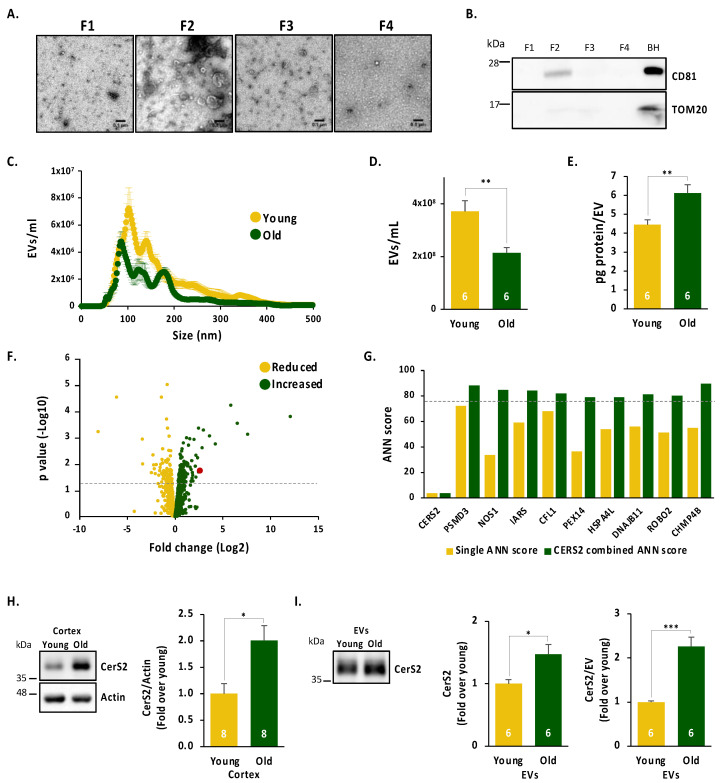
Characterisation of EVs isolated from young and old cortices: (**A**,**B**) electron microscopy (**A**) and WB (**B**) studies of the different fractions of the sucrose gradient phases showing EVs are found in fraction 2. (**C**,**D**) Nanosight analysis of young and old EVs from cortical regions showing the distribution size (**C**) and the number of total EVs (**D**) showing that the total number of EVs is reduced in the ageing cortex. (**E**) Quantification of protein per EV in cortical EVs from young and old cortices shows that aged EVs present higher protein levels. (**F**) Volcano plot of proteins up- and downregulated in aged cortical EVs. CerS2 is indicated in red. The dotted line indicates *p*-values < 0.05. (**G**) Bioinformatic analysis based on artificial neural networks (ANN) for the synergic effect of CERS2 and different proteins predicted in relation to apoptosis. An ANN scores greater than 78 predicts some function in apoptosis. The dotted line indicates scores above 78. (**H**) Representative images for Western blot (**left**) and quantification (**right**) of CerS2 in young and old mice cortex homogenates showing increased total levels of CerS2 in the ageing cortex. (**I**) Representative images for Western blot (**left**) and quantification for CerS2 (**middle**) and CerS2 per EV (**right**) in young and old mice cortex-derived EVs showing increased levels of CerS2 and CerS2 levels per EV. Student *t*-test. * *p*-value < 0.05, ** *p*-value < 0.01, *** *p*-value < 0.001.

Changes in intercellular communication are one of the hallmarks of ageing [28], also evident in the ageing brain [29]. Among the various mechanisms of intercellular communication, one that has gained special attention in recent years is that mediated by EVs. Therefore, to determine the effect of ageing on brain intercellular communication, we purified EVs from the cerebral cortex of young and old mice. As shown in Figure 1, fraction 2 of the buoyant sucrose gradient shows enrichment for EVs when determined by EM (Figure 1A), which is positive for the EVs marker CD81 and negative for the mitochondrial protein TOM20 (Figure 1B). After confirming the proper purification of EVs, we studied the effect of age on EVs’ size and number. Nanotracking analysis revealed that the overall size of the vesicles is not significantly affected by age (size varied between 50–500 nm), yet the number of vesicles is reduced in old mice (Figure 1C,D). Given the different size distributions of aged cortical EVs, and to account for the reduced number of vesicles found in the old cortex, we further studied EVs’ size by normalising them to their numbers and expressing them in percentages (Supplementary Figure S1). However, cumulative frequency analysis of EVs’ size expressed in percentage did not show any significant changes (Supplementary Figure S1). Finally, we wondered whether the protein content per EV is altered in the ageing cortex. Our results showed that ageing leads to increased protein content per cortical EV when analysed by protein concentration corrected per EV concentration of a given sample (Figure 1E). Therefore, we conclude that the EV number is reduced but loaded with higher amounts of protein in the ageing cortex.

The previous result led us to investigate which proteins in cortical EVs are altered with age. To this end, fraction 2 containing EVs from young and old cortices was analysed by mass spectrometry. Of the 2806 identified proteins (Supplementary Table S1), 429 showed quantitative differences between old and young mice, of which 151 were upregulated and 278 downregulated (Figure 1F). Given the very high number of proteins up- and downregulated in aged EVs, we conducted a bioinformatic analysis based on artificial neural networks (ANN). Briefly, this analysis generates a mathematical model which mimics the complex behaviour of candidate proteins in the brain’s ageing processes based on bibliography and databases. Our resulting ANN analysis predicted a number of cellular functions altered in aged EVs, including oligodendrocytes and myelin (Supplementary Figure S1). Given that oligodendrocyte-to-neuron exosome transfer promotes neuronal long-term maintenance by facilitating axonal transport [21], we wondered which oligodendrocytic proteins were specifically altered in our proteomic study. Our results show that four oligodendrocyte proteins are enriched in EVs from the aged cortex, which presented significant (or close) fold changes: ceramide synthase 2 (CerS2), myelin basic protein (MBP), myelin proteolipid protein (PLP1) and myelin oligodendrocyte glycoprotein (MOG). Of these, the enzyme with the highest increased levels with age was CerS2 (Supplementary Table S1).

CerS2 has been mainly described as responsible for the synthesis of very long-chain (VLC) ceramides that are incorporated into galactosylceramide (GalCer), an essential component of myelin. In fact, mice lacking CerS2 exhibit a 50% loss of compact myelin [30] and mice lacking the enzyme required for GalCer synthesis (ceramide galactosyltransferase, CGT) are unable to synthesise GalCer and sulfatide, resulting in myelin instability, conduction deficits, tremor, hindlimb paralysis and premature death [31,32]. Several studies controversially link the activity of CerS2 to the inhibition/activation of apoptosis/survival pathways [33,34,35,36,37,38]. Interestingly, the apoptosis function is also predicted from the ANN analysis (Supplementary Figure S1). In addition, the ANN scores are higher for proteins identified for the apoptosis function when analysed with a synergic effect with CerS2 (Figure 1G), suggesting that CerS2 favours the role of each protein in apoptosis.

In order to validate our proteomic data, we measured the levels of CerS2 by Western blotting. Our results showed increased total levels of CerS2 in cortical homogenates from aged animals (Figure 1H). Note that increased CerS2 protein levels seem not to be a transcriptional event as increased CerS2 mRNA levels are not observed in the aged brain when analysed by single-cell RNAseq [39]. Next, we evaluated the levels of CerS2 in cortical EVs from young and old animals. We found increased CerS2 levels in the aged cortical EVs (Figure 1I), which is even more evident when normalised to the number of EVs per sample (Figure 1I). Together, these results show that the size of cortical EVs does not change in the ageing cortex but that their number and content are affected, including high levels of the oligodendrocytic protein CerS2.

### 2.2. CerS2 Is Present in Oligodendrocytes and in Cortical Neurons In Vivo

**Figure 2:**
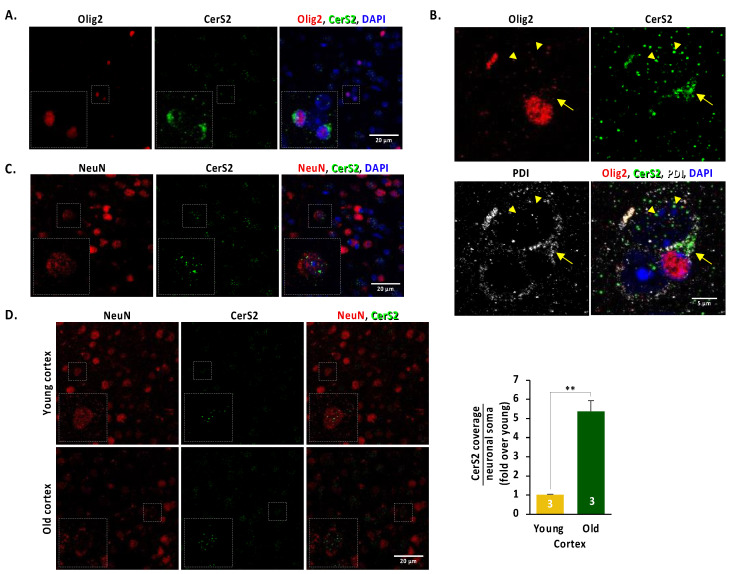
Immunolocalisation analysis of CerS2 of the mice brain cortex. (**A**) Perinuclear localisation of CerS2 in Olig2 positive cells showing CerS2 is expressed in oligodendrocytes. (**B**) Colocalisation of CerS2 with the endoplasmic reticulum (ER) marker PDI and Olig2 positive oligodendrocytes. Triangles point to a punctuate pattern in nuclei and perinuclear area resemblant to the one found in neurons. Arrow points to CerS2 diffuse pattern that colocalises with PDI perinuclear staring typical of ER. (**C**) CerS2 punctuate pattern in NeuN positive neurons. (**D**) Analysis of CerS2 punctuate pattern area per neuronal soma in the young and old cortex shows increased CerS2 levels in the aged neurons. Student *t*-test. ** *p*-value < 0.01.

CerS2 is synthesised by oligodendrocytes [40,41], and given we show CerS2 enrichment in EVs from the aged cortex, we hypothesised that one of the targets of these vesicles could be neurons, where they could participate in the “tug-of-war” between apoptosis and survival forces. Consistent with this possibility, in situ immunolocalisation analysis of CerS2 revealed a positive signal in both oligodendrocytes and neurons. In oligodendrocytes (Olig2 positive cells) CerS2 shows a juxtanuclear staining pattern, typical of de novo synthesised membrane protein (Figure 2A), co-localising with the protein disulfide isomerase (PDI) folding assistance chaperone (Figure 2B). In neurons (NeuN positive cells) the pattern is finely punctuated in the cell body, but not in the endoplasmic reticulum (Figure 2C). Importantly, the punctuated pattern in neurons increases with age (Figure 2D). Thus, our result shows that CerS2 is present in both oligodendrocytes and neurons with a very different pattern and that the neuronal punctate pattern is increased in the aged cortex.

### 2.3. Neuronal CerS2 Originates from Oligodendrocyte-Derived EVs

**Figure 3:**
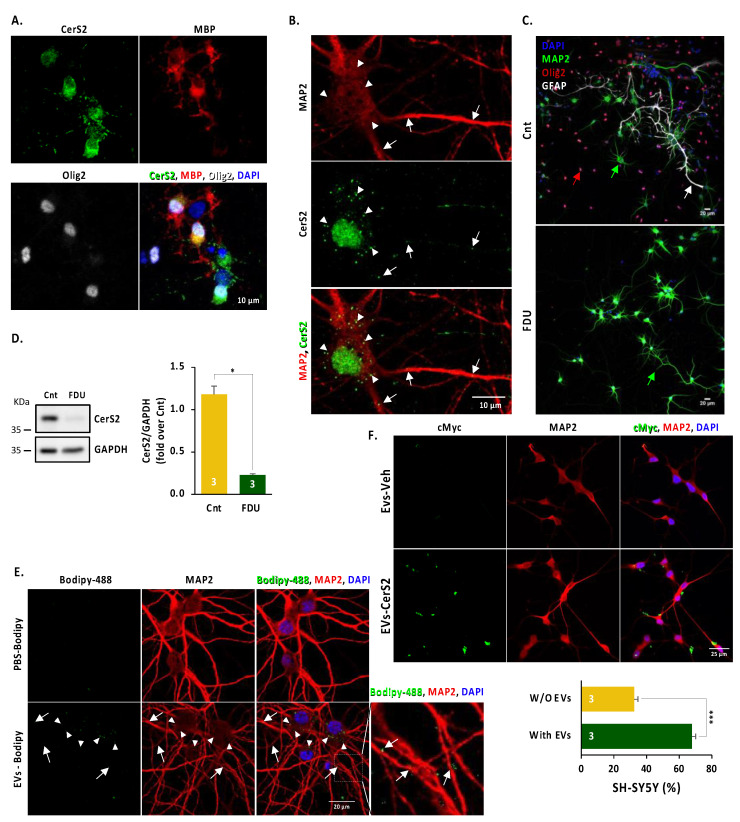
Neuronal CerS2 originated in oligodendrocytes and can be transferred to neurons via EVs. (**A**) Immunolabelling analysis for CerS2 localisation in oligodendrocytes in cortical cultures shows that CerS2 is expressed perinuclearly in mature oligodendrocytes (Olig2 and MBP-positive cells) present in the culture. (**B**) Immunofluorescent analysis for CerS2 localisation in cortical neuronal cultures labelled with MAP2 shows that CerS2 present a punctuate pattern localised in the neuronal somas (triangles) and neurites (arrows). (**C**) Images of 7DIV neuronal cultures treated with vehicle (Cnt) or FDU showing the removal of non-neural cells. Arrows indicate neurons (green), oligodendrocytes (red) and astrocytes (white). (**D**) Representative Western blot image (**left**) and quantification (**right**) of CerS2 in vehicle (Cnt) and FDU-treated neuronal cultures showing a dramatic reduction in CerS2 levels. (**E**) Representative images for cortical primary cultures treated with 1.3×10^8^ EVs/mL Bodipy (PBS-Bodipy) or EVs-labelled with Bodipy (EVs-Bodipy) showing the neuronal uptake of oligodendrocyte-derived EVs. CerS2 presents a punctuate pattern localised in the neuronal somas (triangles) and neurites (arrows). (**F**) Representative images (upper panels) and quantification (bottom panel) for differentiated SH-SY5Y treated with 1.3×10^9^ EVs/mL vehicle (EVs-Veh) and CerS2-loaded (EVs-CerS2) EVs neuronal uptake of CerS2 (cMyc) showing that EVs-CerS2 is up taken by differentiated SH-SY5Y. Student *t*-test. * *p*-value < 0.05, *** *p*-value < 0.001.

To determine if neuronal CerS2 originates from oligodendrocytes, we performed a series of experiments in primary cultures of rodent embryonic cortex. Although most of the cells present in these cultures correspond to neurons, oligodendrocytes and astrocytes are also commonly found. Figure 3A shows that the oligodendrocytes present in the culture (positive for MBP) show strong somatic staining for CerS2, while the neurons (cells positive for MAP2) show uniformly distributed puncta in the cell body and neurites (Figure 3B), reminiscent of the pattern found in vivo (Figure 2). To test if neuronal CerS2 originates from the oligodendrocytes present in the culture, we eliminated all non-neuronal cells present in the culture by adding 5-fluorodeoxyuridine (FDU) to the medium [42,43], an inhibitor of nucleic acid and protein synthesis. Our results show that the treatment cleared the culture of non-neuronal cells as reflected by the absence of Olig2 and GFAP-positive cells (Figure 3C). Furthermore, the treatment led to a significant reduction in CerS2 levels (Figure 3D). These results confirm that CerS2 is produced by oligodendrocytes and suggest that the CerS2 puncta in neurons could represent enzyme transferred from oligodendrocytes, probably via EVs. To test this hypothesis, we first analysed whether exosomes from oligodendrocytes can be taken up by neurons. We isolated EVs from the medium of rodent primary oligodendrocytes in culture (Supplementary Figure S2) and confirmed the procedure by electron microscopy (Supplementary Figure S2). Next, in order to analyse the neuronal uptake of EVs, we labelled EVs from oligodendrocytes with the lipophilic compound Bodipy, allowing us to fluorescently track oligodendrocyte-derived EVs (Supplementary Figure S2). Adding the purified EVs labelled with Bodipy to neuronal cultures revealed Bodipy fluorescent puncta in neuronal cell bodies and neurites (Figure 3E), strengthening the notion that oligodendrocytes communicate with neurons via EVs.

To directly assess if oligodendrocyte-derived EVs are the source of CerS2 present in neurons, we purified exosomes from the human oligodendrocyte HOG cell line and used them to treat differentiated human neuron-like SH-SY5Y cells. First, we generated a HOG stable line expressing CerS2-cMyc (HOG-CerS2, Supplementary Figure S2). Next, we purified exosomes from HOG-CerS2, which were validated by electron microscopy and indeed were loaded with CerS2-cMyc when analysed by Western blotting (Supplementary Figure S2). Finally, HOG-CerS2-derived EVs were added to differentiated SH-SY5Y (Supp Figure S2), allowing us to trace EVs containing CerS2. Our results show that 67.68% of the differentiated SH-SY5Y cells showed positive cMyc staining after the addition of HOG-derived CerS2-containing EVs (Figure 3F). Efficient internalisation of EVs from the HOG-expressing CerS2-cMyc was also observed in HEK cells (Supplementary Figure S3). Taken together, our results show that oligodendrocyte-derived EVs containing CerS2 are taken up by neurons and that this intercellular communication is enhanced during ageing.

### 2.4. Inflammatory Stimuli Lead to Increased CerS2, Which Contributes to Neuronal Survival When Present in EVs

**Figure 4:**
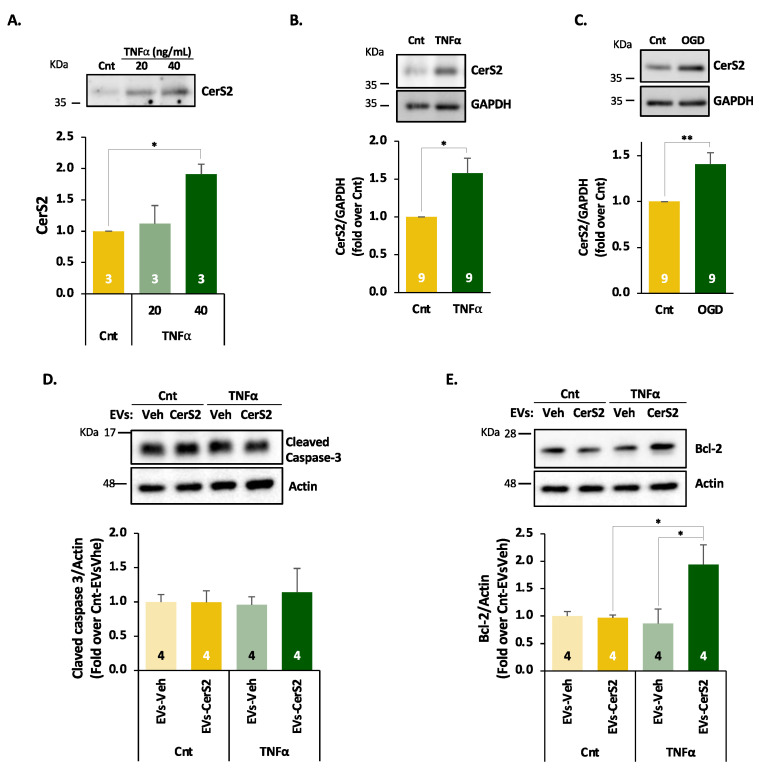
CerS2 expression is induced in an inflammatory context leading to the expression of antiapoptotic factors. (**A**) Representative image (top) and quantification (bottom) for CerS2 levels in EVs secreted to the medium of cortical cultures upon vehicle (Cnt) and TNF⍺ (20 and 40 ng/mL) treatment showing an increase in CreS2 level in secreted EV with 40 ng/mL of TNF. (**B**,**C**) Representative images (**top**) and quantification (**bottom**) for CerS2 levels in total lysates from neuronal cultures challenged with TNF⍺ (**A**) or subjected to metabolic stress (**B**, OGD) showing an increase in CreS2 levels. (**D**,**E**) Representative images (**top**) and quantification (**bottom**) for cleaved caspase-3 (**D**) and Bcl-2 (**E**) in differentiated SH-SY5Y treated for 24 hours with vehicle (Veh) or CerS2-loaded EVs (CerS2) in the presence or absence of an inflammatory challenge (TNF ⍺). Our results show that mild inflammatory challenges or EVs do not induce apoptosis in differentiated SH-SY5Y but that CerS2-loaded EVs lead to increased antiapoptotic factors (Bcl-2) in an inflammatory context. One-sample *t*-test (**B**,**C**) and ANOVA followed by Tukey post hoc (**A**,**D**,**E**). * *p*-value < 0.05, ** *p*-value < 0.01.

We next questioned whether the increased expression of CerS2 in the old brain was related to age-typical alterations. Given that increased brain inflammation is another clear sign of ageing [44] and that the pro-inflammatory cytokine tumour necrosis factor-α (TNFα) is upregulated in the ageing brain [45], we induced an inflammation-like state by adding increasing concentrations of TNFα to primary rodent cortical neurons [46,47]. Figure 4A shows that TNFα led to a significant increase in CerS2 levels in secreted EVs purified from the medium, which also induced a concomitant increase in the total number of EVs (Supplementary Figure S4) as previously described [48,49]. Next, we wonder if increased CerS2 EV levels upon TNFα challenge were due to increased synthesis of CerS2. Our results showed that TNFα indeed upregulates total CerS2 levels in total cell homogenate (Figure 4B). A similar increase was evident when the primary cultures were subjected to metabolic stress, by means of nutrient deprivation (Figure 4B). Taken together, these results suggest that increased CerS2 levels in cortical neurons and in EVs during ageing (Figure 1) is the result of the chronic inflammatory state of the ageing brain.

In oligodendrocytes, CerS2 is important for the synthesis of myelin sphingolipids. However, the role of Cers2 in neurons remains elusive. Nonetheless, studies have shown that the expression of CerS2 rescues the neurodegeneration phenotype resulting from the lack of the neuronal ceramide synthase 1 (CerS1) [50], suggesting a possible pro-survival role. Given that we found apoptosis as a cellular function possibly affected by aged cortical EVs in our ANN analysis (Supplementary Figure S1), we analysed the levels of the apoptotic marker cleaved caspase-3 and the anti-apoptotic factor Bcl2 in differentiated SH-SY5Y treated with control or CerS2-loaded EVs from HOG-CerS2 and in the presence or absence of TNFα. Our results showed that 24 h treatment of a mild inflammatory insult or the presence of control/CerS2 EVs do not activate apoptosis as no changes in cleaved caspase-3 were observed (Figure 4D). Interestingly, HOG-derived EVs containing CerS2 lead to the upregulation of the anti-apoptotic factor Bcl-2 only in an inflammatory context (Figure 4E), thus suggesting a neuroprotective role of CerS2-loaded oligodendrocytes-derived EVs.

## 3. Discussion

Age is accompanied by structural alterations of the brain, both of grey matter (reduction of the dendritic tree, shrinking of the soma, fewer synapses) and of white matter (reduction of myelin, the appearance of periaxonal alterations, myelin degradation) [51,52]. Despite these alterations, cognitive deficits are relatively minor and in general, do not prevent older individuals from living a full life. The capacity to function despite the numerous structural alterations (and the underlying biochemical changes) is the result of the most robust mechanisms for damage repair that neurons engage to fight against the noxious stimuli occurring in ageing. In addition to the cell’s autonomous repair mechanisms, survival is also nourished by stimuli that come from other cells, either through electrical stimuli, soluble factors or extracellular vesicles. Our study reflects the importance of the latter as we show that old cortical neurons incorporate CerS2 from oligodendrocyte-derived EVs, in turn favouring neuronal resilience to inflammatory effects agents such as TNFα. Specifically, we have shown (i) age-associated increased levels of the very long-chain ceramide synthase CerS2, which have an oligodendrocytic origin [40], (ii) that CerS2 is present in oligodendrocyte-derived EVs and that CerS2 levels increase in aged cortical EVs, (iii) that a small but significant amount of CerS2 is present in neurons, possibly as a consequence of neuronal uptake of oligodendrocytes-derived EVs, (iv) that the amount of CerS2 in neurons increases during ageing, (v) that inflammatory and stress stimuli increase CerS2 levels in cortical neurons and EVs and (vi) that oligodendrocytic EVs loaded with CerS2 favours neuronal antiapoptotic process in an inflammatory context.

In the brain, CerS2 is expressed in oligodendrocytes. CerS2 catalyses the synthesis of very long acyl chain ceramides, which generate sphingomyelin after the addition of phosphocholine. This implies CerS2’s main role in the brain is to contribute to the generation of myelin. Supporting this idea, transgenic mice in which CerS2 or CGT coding region has been inactivated show a 50% loss of compacted myelin, 80% loss of myelin basic protein [30] and impaired GalCer and sulfatide synthesis [31,32], all of which are required for myelin synthesis and normal brain development. In addition, CerS2 and its metabolites are associated with Alzheimer’s disease (AD) [53] and CerS2 activity is reduced in early AD stages, leading to the demyelination observed in the disease [54]. Nevertheless, since ceramides are an essential component of the plasma membrane, where in addition to their structural role they play a role in signalling, it cannot be ruled out that the increase in CerS2 in the brain with age has other functions besides contributing to myelin synthesis. In this regard, some studies awarded CerS2 a role as a pro-survival [33,34,35] although others as a pro-apoptotic [36,37,38]. The discrepancy may be due to different factors, a critical one being the levels of the different products of CerS2 activity, which may contribute to signalling towards death or survival. Regardless, in the context of our experimental system, our results are more in accordance with the data suggesting a pro-survival role of CerS2 in neurons, as EV loaded with CerS2 led to increased levels of Bcl-2 in a pro-inflammatory context. We do not know the mechanistic link between high CerS2 and activation of Bcl-2, but it does not seem unreasonable to postulate that it is part of the machinery activated when there is an increase in sphingolipids. In fact, numerous studies have shown that sphingolipids influence multiple aspects of cells survival/death equilibrium, either via the Akt/protein kinase B signalling, a pathway that regulates metabolism, stress response and the activity of the anti-apoptotic Bcl-2 family of proteins, via transcription factors including NF-κB, FOXOs and AP-1 and even by regulating exosomes and other secretion mechanisms [55]. However, we cannot rule out that part of the pro-survival effects mediated by oligodendrocyte-derived EVs with high CerS2 content are a result of an effect on other survival pathways mediated by other components present in the same vesicles. In any case, we can conclude that oligodendrocyte-derived EVs have a positive effect when taken up by neurons. This conclusion follows previous work showing that oligodendrocyte-derived EVs can exchange oligodendrocyte-synthesised proteins such as PLP1 and CNP (myelin-related as CerS2) with neurons, contributing to neuron homeostasis and metabolic activity [21].

Another aspect of our results worth discussing is the cause behind the increase in CerS2 during ageing. As we age, inflammation increases in our brains, mainly due to an increase in the reactivity of microglia. Microglia are the primary source of proinflammatory cytokines, including TNFα, inducing or modulating a broad spectrum of cellular responses, positive and negative. As the brain ages, the levels of TNFα increase [45]. However, this does not necessarily mean a deleterious effect, as TNFα is mostly a pleiotropic cytokine, capable of orchestrating different types of cellular responses, including neuronal protection against toxic agents [56]. On the other hand, TNFα can also mediate apoptosis, and it has been implicated in the pathogenesis of a wide spectrum of human diseases [57,58,59]. Irrespective of what the increase in TNFα does in the ageing brain, what its elevated presence implies is that brain cells are in a chronic inflammation state and therefore must be protected from it. We here show that TNFα can trigger a pro-survival mechanism in the old brain, as it increases the synthesis of CerS2. As we have seen, Cers2 is sent to extracellular space throughout EVs, which are then taken up by neurons (and possibly other brain cells) and promote a survival response. Naturally, we do not mean that TNFα is playing this unique role in the old brain. As has already been shown on numerous occasions, whether a molecule performs beneficial or negative functions are related to its levels, location or circumstances. As we are yet to mechanistically understand how oligodendrocyte-derived EVs containing CerS2 activate survival, we are also unaware of the mechanism behind the upregulation of CerS2 by TNFα. However, the extensive research conducted by the Wagner group on the regulation of ceramide synthase may offer some insight into this phenomenon [60].

Taken together, our study shows for the first time that ageing affects intercellular communication in the brain as seen by the reduced number of EVs. Interestingly, increased levels of the ceramide synthesis enzyme CerS2 are found in aged cortical EVs, which are produced by oligodendrocytes and transferred to neurons, boosting neuronal survival.

## 4. Materials and Methods

### 4.1. Experimentation Animals

All procedures involving animals were conducted according to European 2010/63/UE) and Spanish (RD 53/2013) legislation, in compliance with the ethical standards Centro de Biología Molecular Severo Ochoa and performed under an authorised project licence (PROEX 204/19). C57BL6/J mice and Wistar rats were used in this study. Animals were kept in ventilated racks at 22 ± 2 °C and 55 ± 10% humidity with a 12 h/12 h light cycle and free access to food and water at all times.

### 4.2. Primary Cortical Neurons Cultures

Primary cortical neuron cultures were obtained from embryonic brains at day 18 (E18) of pregnant Wistar rats. Cortices were obtained from both hemispheres, which were dissected in cold Hank solution (0.45% glucose (G8769-100ML, Sigma-Aldrich, St. Louis, MO, USA) and 7 mM HEPES (#H0887, Sigma-Aldrich, St. Louis, MO, USA)) and followed by enzymatic digestion (0.03% Trypsin (#11500636, Invitrogen, ThermoFisher Scientific, Waltham, MA, USA) and 72 mg/mL DNAase (#10104159001, Sigma-Aldrich, St. Louis, MO, USA)) at 37 °C for 16 min. Cortices were washed three times with plating medium (MEM medium + 10% inactivated horse serum (#16050122, Gibco, ThermoFisher Scientific, Waltham, MA, USA) + 20% glucose), dissociated in 5 mL of plating medium and passed through a 70 µM cell strainer. Cells were counted and plated in plating medium onto plates W/O coverslips precoated poly-L-lysine and kept in standard culturing conditions (37 °C and 5% CO_2_ in a humidified incubator) for 4 h. Subsequently, plating medium was replaced with pre-equilibrated Neurobasal medium supplemented with B27 and GlutaMAX. After 7 DIVs, medium was replaced with neurobasal medium supplemented with B27 without GlutaMAX. To induce inflammatory stress, primary neuronal cultures were treated with 20 or 40 ng/mL of tumour necrosis factor-α (TNFα, #315-01A Peprotech, ThermoFisher Scientific, Waltham, MA, USA), once every 24 h, for a total of 48 h. For metabolic stress, primary cultures were kept in oxygen–glucose deprivation (OGD) medium (1 mM CaCl_2_ × H_2_O_2_, 5 mM KCl, 137 mM NaCl, 0.4 mM KH_2_PO_4_, 0.3 mM Na_2_HPO_4_ × 12H_2_O, 0.5 mM MgCl_2_ × 6H_2_O, 0.4 mM MgSO_4_ × 7H2O, 25 mM HEPES, 4 mM NaHCO_3_ final pH 7.3) for 6 h. Neuronal cultures were treated with 1.3 × 10^8^ EV particles/mL extracted from primary oligodendrocytes and labelled with Bodipy for 24 h. To remove dividing cells (mainly oligodendrocytes and astrocytes), cultures were treated with 1 µM of 5-Fluoro-2′-deoxyuridine (FDU, #856657, Sigma-Aldrich, St. Louis, MO, USA) on day 2 for 24 h when the medium was replaced with fresh neurobasal supplemented medium.

### 4.3. Primary Oligodendrocyte Culture

A total of 6 × C57BL6/J pup brains were dissociated at P6 following the protocol for the “Neural Tissue Dissociation” kit (#130-092-628, Miltenyi Biotec GmbH, Germany). Briefly, cortices were enzymatic digestion with papain in Hank’s solution at 37 °C for 15 min and cells were dissociated. Finally, oligodendrocyte precursors were labelled with antibodies against A2B5 bound to magnetic beads and purified using Ms Columns kit columns (#130-042-201, Miltenyi Biotec GmbH, Germany) following the kit instructions. Purified oligodendrocytes were counted and seeded onto plates precoated with 0.1mg/mL poly-L-lysine (#P2636, Sigma-Aldrich, St. Louis, MO, USA) and 10 μg/mL laminin (L-2020, Sigma-Aldrich, St. Louis, MO, USA), in MACS Neuromedium (#130-093-570, Miltenyi Biotec GmbH, Germany), supplemented with 10% MACS neurobrew-21 (#130-093-566, Miltenyi Biotec GmbH, Germany), 100 U/mL penicillin–streptomycin (#11548876, Gibco, ThermoFisher Scientific, Waltham, MA, USA), 2 mM glutamine (G7513, Sigma-Aldrich, St. Louis, MO, USA), 1 mM glucose (G8769, Sigma-Aldrich, St. Louis, MO, USA), 1 ng/mL PDGFα (#P3076, Sigma-Aldrich, St. Louis, MO, USA) and FGF-2 (#SRP4038, Sigma-Aldrich, St. Louis, MO, USA). Primary oligodendrocytes were cultured in standard culturing conditions (37 °C and 5% CO_2_) for two weeks and the medium was collected and replaced every other day for the EV extraction.

### 4.4. SH-SY5Y Cell Line

Cells were cultured in DMEM medium + 10% foetal bovine serum (FBS) (#10270-106, Gibco, ThermoFisher Scientific, Waltham, MA, USA), at 37 °C in a humidified incubator containing 5% CO_2_. To induce differentiation, SH-SY5Y were plated onto coverslips/plates precoated with rat tail collagen (A1048301, Gibco, ThermoFisher Scientific, Waltham, MA, USA), and differentiation was induced by replacing culture medium for neurobasal medium (#2110303049, Gibco, ThermoFisher Scientific, Waltham, MA, USA) supplemented with B27 (#11530536, Gibco, ThermoFisher Scientific, Waltham, MA, USA), GlutaMAX—(#35050-061, Gibco, ThermoFisher Scientific, Waltham, MA, USA) and 10 µM retinoic acid (RA) (302-79-4, Merck, Burlington, MA, USA). The medium was replaced with fresh medium three days after. At day five, the medium was replaced with neurobasal supplemented with B27, GlutaMAX and BDNF (50 ng/mL, #450-02, Peprotech, ThermoFisher Scientific, Waltham, MA, USA) and cells were used three days after. To induce inflammatory stress, differentiated SH-SY5Y cells were treated with 20 ng/mL of tumour necrosis factor-α (TNFα, #315-01A Peprotech, ThermoFisher Scientific, Waltham, MA, USA) for 24 h in the presence of 1.314 × 10^9^ EV particles/mL extracted from HOG-Veh/HOG-CerS2 cell lines.

### 4.5. HOG Cell Line

Cells were cultured in low glucose (1 g/L) DMEM medium + 10% FBS under standard culturing conditions (37 °C and 5% CO_2_ in a humidified incubator). HOG cell line was used to generate a stable line expressing CerS2 (HOG-CerS2, see “Generation of HOG-CerS2 line”), which was used for obtaining EVs containing CerS2. For EVs purification, HOG-CerS2 cells were plated in 15 cm plates with 20 mL of DMEM + 10% FBS previously depleted of vesicles (100,000× *g* centrifugation for 16 h). When the cells reached 50% confluence, the medium was replaced with DMEM + 5% FBS. After 3 days, the medium is collected and stored for EVs’ purification.

### 4.6. Generation of HOG-Veh and HOG-CerS2 Lines

To generate a monoclonal stable HOG line expressing CerS2 (HOG-CerS2) we first produced empty lentivirus carrying a Puro resistance cassette (Veh, PS100092, Origene, Rockville, MD, USA) or carrying the overexpression of the Rat CerS2 followed by a P2A cleaving sequence and the Puro resistance cases (CerS2, RR202144L3, Origene, Rockville, MD, USA). Lentiviral particles were produced in HEK-293T cells using the aforementioned plasmids and packaging plasmids CMV and MD2.G and titrated as we have previously [61]. To generate a monoclonal HOG-CerS2 and empty vector control (Veh) cell lines, 100,000 HOG cells were plated in 3 cm dishes and cultured with low glucose (1 g/L) DMEM + 10% FBS throughout the cell line generation process. HOG cells were infected with Veh or CerS2 containing lentiviral particles 24 h after plating. The next day, HOG cultures were treated with 4 µg/mL of puromycin for 24 h to positively select HOG-infected cells. HOG cells that were positively selected were grown to confluency. Monoclonal isolation was achieved after plating serial dilutions in 96-well plates to obtain 1 cell/well. Finally, clones were grown and screened for CerS2-cMyc by Western blot and immunofluorescence.

### 4.7. HEK Cell Line

HEK cell line was obtained form ATCC (CRL-3216). Cells were cultured in low glucose (1 g/L) DMEM medium + 10% FBS under standard culturing conditions (37 °C and 5% CO^2^ in a humidified incubator). 

### 4.8. Isolation of EVs from Mouse Cerebral Cortex Tissue

Cerebral cortex-derived EVs were isolated following the protocol described by Vella and colleagues with minor modifications [62]. Briefly, mice were deeply anaesthetised with a ketamine/xylazine and transcardially perfused with cold saline (0.9% sodium chloride). Brain tissue was collected and stored at −80 °C until use. Cortical pieces of about 2 mm^2^ were incubated at 37 °C for 10 min in hibernate medium (#A12476-01, Gibco, ThermoFisher Scientific, Waltham, MA, USA) containing collagenase (75 U/mL, #LS004206, Worthington) at a ratio of 800 µL of solution per 100 mg of tissue. Samples were gently homogenised with a 10 mL pipette (5 strokes) and incubated at 37 °C for 5 min more and placed on ice when protease (#04693132001, Roche, Switzerland) and phosphatase (#524627, Merck Millipore, Burlington, MA, USA) inhibitors were added. Next, samples were centrifuged at 300× *g* for 5 min at 4 °C. The supernatant was recovered and centrifuged at 2000× *g* for 10 min at 4 °C and the recovered supernatant was centrifuged again at 10,000× *g* for 30 min at 4 °C. Next, the recovered supernatant (F1) was laid on a sucrose gradient (1.2 mL of 0.6 M sucrose (F2), 1.2 mL of 1.3 M sucrose (F3) and 1 mL of 2.5 M sucrose (F4)) and centrifuged at 180,000× *g* for 3 h at 4 °C in an ultracentrifuge rotor SW40 (Beckman Coulter, Brea, CA, USA). The different fractions (F1–F4) were recovered, washed with 13 mL of cold-filtered phosphate-buffered saline (PBS) and centrifuged at 100,000× *g* for 1 h at 4 °C in a swinging rotor (TST28.38, Beckman Coulter, Brea, CA, USA). Finally, protein pellets were resuspended in 38 µL of cold-filtered PBS. EVs were analysed by nanoparticle tracking analysis using a NanoSight NS500 device (Malvern Panalytical).

### 4.9. Isolation of EVs from Cell Culture Medium

Medium collected from primary oligodendrocyte cultures or HOG cell lines were centrifuged to remove cell debris, first at 200× *g* for 10 min at 4 °C and then the supernatant was recovered and centrifuged at 2000× *g* for 10 min at 4 °C. Supernatants were recovered and stored at −80 °C until use. To remove larger apoptotic bodies and vesicles, supernatants were centrifuged at 10,000× *g* for 30 min at 4 °C in a swinging rotor (TST28.38, Beckman Coulter, Brea, CA, USA). Subsequently, the recovered supernatants were placed onto 4 mL of fraction buffer (30% sucrose) and centrifuged for 1.5 h at 100,000× *g* at 4 °C. Next, the bottom 6 mL containing 2 mL of medium, and 4 mL of fractionation buffer were collected, washed with 30 mL of cold-filtered PBS and centrifuged at 100,000× *g* for 1.5 h at 4 °C. Pellet containing EVs were resuspended in 400 µL of sterile PBS and filtered. EVs were analysed using a NanoSight NS500 (Malvern Panalytical, UK).

### 4.10. Labelling Exosomes with Bodipy

EVs isolated from mouse oligodendrocyte cultures’ medium were labelled using a lipophilic dye, Bodipy 493/503 (4,4-difluoro-1,3,5,7,8-pentametil-4-bora-3a,4a-diaza-s-indaceno, #D3922, Thermofisher Scientific, Waltham, MA, USA). Briefly, PBS containing EVs were incubated with 1 μM Bodipy in PBS for 1 h at 37 °C in 5% CO2. Unincorporated dye was removed using size exclusion columns (MW 3000, #4484449 ThermoFisher Scientific, Waltham, MA, USA) following the manufacturer’s instructions. We subjected PBS without containing EVs to the same procedure and used it as a control. Labelled EVs were immediately used.

### 4.11. Protein Identification by Western Blot

Cell samples, brain cortex tissue or EVs were homogenised in RIPA lysis buffer (20 mM Tris–HCl, pH 7.5, 150 mM NaCl, 1 mM EDTA, 1 mM EGTA, 1% NP-40, 1% sodium deoxycholate and 0.1% SDS) supplemented with protease (#04693132001, Roche, Switzerland) and phosphatase (#524627, Merck Millipore, Burlington, MA, USA) inhibitors and sonicated. Brain samples were centrifuged (10,000 rpm for 10 min at 4 °C) and supernatants were recovered. All samples were stored at −20 °C until used. Protein was quantification using the BCA Protein Assay Kit (#23225, Thermo Fisher Scientific, Waltham, MA, USA) according to the manufacturer’s instructions. Equal amounts of protein were loaded for cell lysates and tissue homogenates. For EVs studies, equal volumes of resuspended EVs were loaded. Protein samples were electrophoretically resolved in 8–12% Tris-HCL gels under denaturing and reducing conditions and transferred to nitrocellulose membranes (#10600002, Amersham, UK). Membranes were blocked in Tween 20-Tris buffer solution (TTBS: 0.1% *v*/*v* Tween 20, 100 mM Tris-HCl, 150 mM NaCl, pH 7.5) containing 5% BSA (Sigma-Aldrich, St. Louis, MO, USA) for 1 h at RT, and incubated overnight at 4 °C with the antibodies (Supplementary Table S2), diluted in 5% BSA in TTBS. Peroxidase-conjugated polyclonal goat anti-rabbit (#P044801-2, Dako, Agilent Technologies, Santa Clara, CA, USA) or anti-mouse-HRP (#P044701-2, Dako, Agilent Technologies, Santa Clara, CA, USA) were used as secondary antibodies at 1:2500 for 1 h at RT in TTBS containing 5% BSA. Bands were visualised with ECL Western Blot Detection Kit (Thermofisher, Scientific, Waltham, MA, USA) in an ImageQuant LAS 4000 Mini (GE Healthcare Life Sciences, Chicago, IL, USA) and quantified with ImageJ.

### 4.12. Immunofluorescence Labelling

For brain samples, mice were deeply anaesthetised with a ketamine/xylazine mixture prior to sequential transcardiac perfusion with cold PBS and 4% paraformaldehyde (PFA) in PBS. After perfusion, brains were collected, fixed in 4% PFA for 24 h hours at 4 °C and subsequently immersed in Glycol for preservation at −20 °C. The brains were then embedded in a 4% agarose and 10% sucrose solution and cut into 40 µm thick sections. Sections were blocked/permeabilised for 1 h with blocking solution (1% BSA and 0.5% Triton X-100 in PBS). For cell cultures, coverslips containing cells were fixed with 4% PFA in PBS for 10 min, permeabilised with 0.1% Triton X-100 in PBS for 5 min and blocked with 1% BSA in PBS at RT. For all immunodetections, primary antibodies (Supplementary Table S2) were incubated in corresponding blocking solution O/N at 4 °C. The next day, sections/coverslips were washed 3 times in PBS, incubated for 1 h with secondary antibodies (Supplementary Table S2) and washed again 3 times in PBS at RT. Finally, sections/coverslips were stained with DAPI (Supplementary Table S2) and mounted onto Superfrost Plus slides (#J1800AMNZ, Epredia, Thermo Fisher Scientific, Waltham, MA, USA) with Mowiol mounting medium. Images were acquired with LSM710 Zeiss or Nikon A1R confocal microscopes and analysed with ImageJ.

### 4.13. Transmission Electron Microscopy

EVs containing samples were placed onto a carbon and formvar grid and stained with 2% uranyl acetate in water for 1.5 min at RT. Grids were washed 3 with distilled water and visualised in a Jem-1010 electron microscope (Jeol, Japan). Images were obtained using 4k × 4k F416 camera (TVIPS).

### 4.14. Proteomics

Proteomics studies were performed by Proteobotics S.L. of the Centro Nacional de Biotecnología (CNB), Universidad Autónoma de Madrid. EVs isolated from the cerebral cortex of young and old mice were run on a 12% SDS-PAGE gel along 1 cm of the separator gel. Subsequently, these bands were stained with colloidal Coomassie blue and were individually cleaved and digested using trypsin. Peptide levels were then quantified by fluorimetry and an amount equivalent to 1 µg of peptides from each digestion was analysed by liquid chromatography (C-18 reverse phase column) coupled to Triple-qTOF mass spectrometer. The data obtained by mass spectrometry was analysed with different search engines such as MASCOT (v.2.5, Matrix Science, UK), OMSSA (v.2.1.9, NCBI, USA), X!Tandem2 (v.win-13-02-01-1, http://www.thegpm.org, accessed 26 July 2018), Myrimatch (v.2.1, Vanderbilt University, Nashville, TN, USA) or MS-GF+ (v.Beta v10072, CCMS-NIGMS, Camden, NJ, USA) and compared versus the Mus musculus database (including Decoy database), in order to obtain the identity of each protein present in EVs samples.

### 4.15. Artificial Intelligence Bioinformatics Analysis

The bioinformatics study using Artificial Neural Networks was performed by Anaxomics Biotech (Barcelona, Spain) using TPMS technology [63]. First, human orthologs of the differentially expressed proteins found in our proteomic of young/old mouse cortical were identified using InParanoid, Mouse Genome Informatics (MGI) and UniProtKB databases. Twenty-five proteins were excluded from the subsequent analysis because of insufficient information. Next, a literature and Gene Ontology database search of identified proteins was performed for physiological functions related to ageing. The cellular processes included mitochondrial dysfunction, oxidative stress, altered Ca2+ homeostasis, dysregulation of synapses and neuronal plasticity, altered DNA repair mechanisms, epigenetic changes, dysregulation of proteostasis and autophagy, processes of increased inflammation (including activation of microglia and astrocytes), dysregulation of apoptosis, dysregulation of glucose uptake, ageing of the vascular system of the brain, disruption of the blood–brain barrier, demyelination and dysregulation of the hypothalamic–pituitary–adrenal axis. A map of the proteins identified around all of the above cellular processes was then generated, adding layers of information including protein–protein interactions, physical interactions and modulations, signalling, metabolic relationships and gene expression. Data for this additional layer of information was collected from databases such as KEGG, BIND, BioGRID, IntAct and REACTOME. Then, the model “was trained” using an input–output table on physiological signalling at the molecular level based on different databases with biological and clinical information on microarrays, protein phosphorylation and drugs. This step generated a list of physiological rules of protein involvement in different processes to build a mathematical model. Subsequently, this mathematical model was processed by artificial intelligence to generate complex networks of protein behaviour in different physiological processes. Finally, the predictive power of the mathematical model is exploited using the artificial neural networks (ANN) strategy [64]. Specifically, each protein is assigned an ANN score and when a particular protein scores equal to or greater than 78, it predicts its involvement in the process of interest, according to its relationship with the set of proteins that represent a given cellular process.

### 4.16. Statistical Analysis

GraphPad Prism 7 (v7, GraphPad Software) was used for statistical analysis. Student’s *t*-test was used for comparisons of two groups. One-sample *t*-test was used to compare groups without standard deviation in the control. One-way ANOVA was used to compare more than two groups and repeated measurements ANOVA to compare repeated measurements from two groups. In the graphs, data are presented as values + SEM and the number of replicates is indicated in the bars. Asterisk indicates *p* values as follows: * *p*-value < 0.05; ** *p*-value < 0.01; *** *p*-value < 0.001.

## Data Availability

Data is contained within the article or supplementary material.

## Supplementary Materials

The following supporting information can be downloaded at: https://www.mdpi.com/article/10.3390/ijms24065986/s1.
