# Supplementary material for: Neuronal Prosurvival Role of Ceramide Synthase 2 by Olidogendrocyte-to-Neuron Extracellular Vesicle Transfer
Source: Int J Mol Sci. 2023 Mar 22;24(6):5986. doi: 10.3390/ijms24065986 (PMC10052063; doi:10.3390/ijms24065986)
Supplement: Supplementary file 1 [file ijms-24-05986-s001.zip › Casadomé-Perales et al_SuppFigs.pdf]

**Supp. Figure S1.**

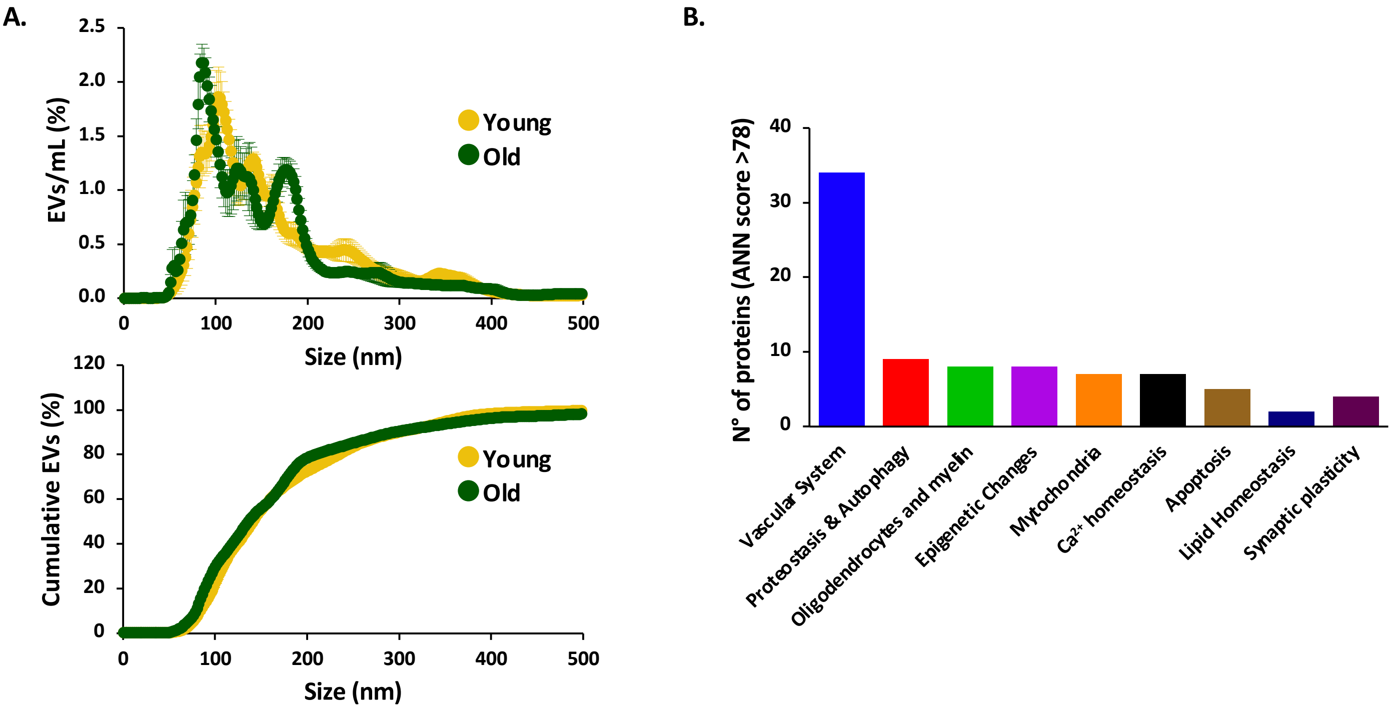


**Supplementary Figure S1. Analysis of cortical EVs from young and old cortex:**
**A)** Analysis of EVs from young and old cortices normalised to the total number of EVs per sample and expressed as %. Graphs show the distribution in % of EVs with different sizes (Top) and the cumulative frequency in % of EVs sizes. **B)** Artificial neuronal network (ANN) prediction for cellular functions affected in ageing based on our EVs proteomic study and the literature. The graph shows the number of proteins (scoring ANN equal to or greater than 78) predicted to be involved in a particular process. Repeated measurement ANOVA (A).

Supp. Figure S2

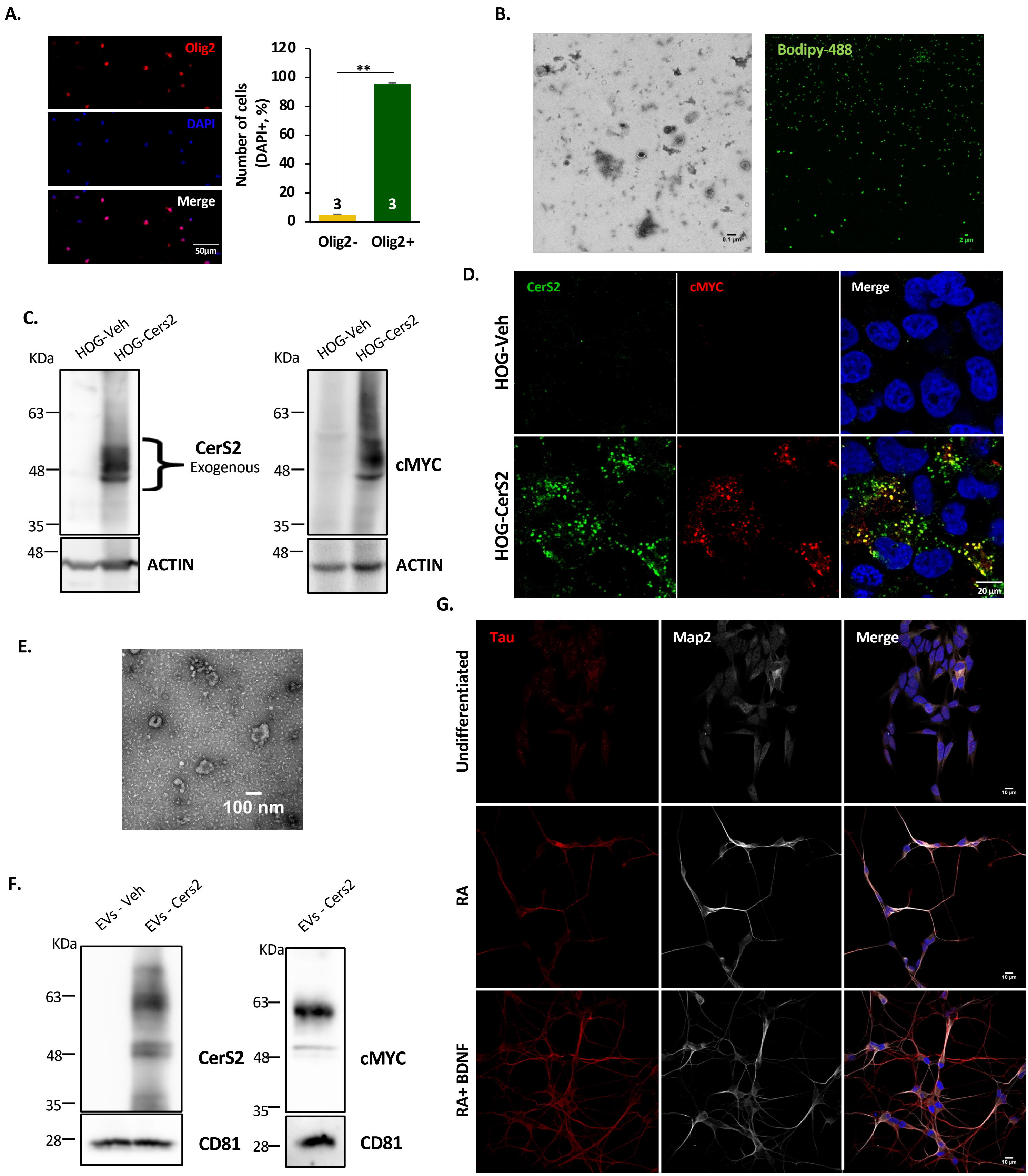


**Supplementary Figure S2. Cellular models validation and derived Evs characterisation:**
**A)** Immunolabeling of primary oligodendrocyte cultures (left panels) and quantification of the percentage of Olig2 positive cells showing that the cultures are highly enriched in oligodendrocytes. **B)** EM images of isolated EVs from primary oligodendrocytes (left) and fluorescence signal of isolated EVs after Bodipy labelling (right). **C-D)** Characterisation of the newly generated HOG cells overexpressing CerS2 (HOG-CerS2) by protein levels using an antibody against CreS2 (B-left) or against the Myc (B-Rigt) and by immunofluorescence (C). Both approaches show the correct overexpression of CerS2 in the HOG-CerS2 line. **E-F)** Representative electron microscopy image (D) and western blots for CerS2 (E-Left) and Myc (E-Right) for EVs isolated from HOG-CerS2. **G)** Representative images for SH-SY5Y differentiation using only retinoic acid (RA, middle panels) or a combination of RA and BDNF (lower panels). SH-SY5Y appear more differentiated as shown per higher arborization and expression of neuronal markers (Tau and MAP2) in the combined treatment of RA+BDNF, the one used for subsequent experiments.

**Supp. Figure S3**

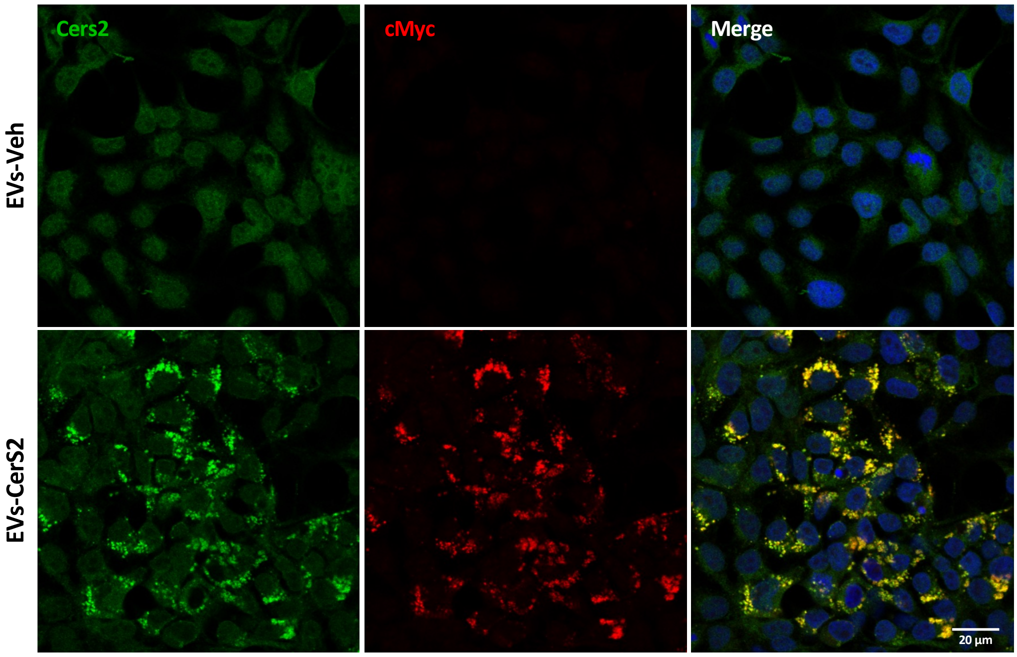


**Supplementary Figure S3. Oligodendrocyte-derived EVs uptake by non-cerebral cells:**
Immunofluorescence of CerS2 in HEK cells treated with control (EVs-Veh) or CerS2-loaded (EVs-CerS2) EVs from HOC-CerS2 line. Our results show that HEK cells also can uptake HOC-derived EV as seen per CerS2 staining in the lower panels.

**Supp. Figure S4**

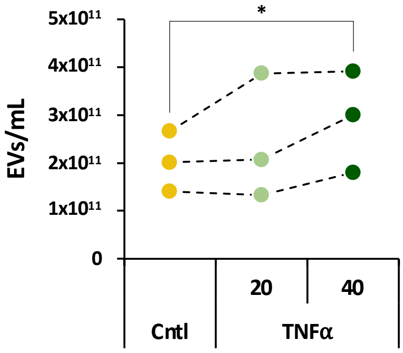


**Supplementary Figure S4. TNFα induced EVs:**
Quantification of the number of EVs in the medium of cortical cultures treated vehicle (Cnt) and TNFα treatments (20 ng/mL or 40 ng/mL) showing an increase of EVs by TNFα. Repeated measurements ANOVA followed by LSD posthoc. t-test. $p<0.05$.
